# 

**Published:** 1887-01-08

**Authors:** 


### THE HOSPITAL

An Institution, Family, and Parochial Journal of Hospitals, Asylums, & all Agencies for the Care of the Sick, Criticism & News.

### CONTENTS.

**Hospital Centralisation at Aberdeen** - 241

**Words of Consolation** - 242

**Who Did It?** By Miss LAURA BAINBRIDGE - 243

**Hospital Funds and the Working Classes.** By Alderman FRED. R. SPARK, Leeds - 245

**The Hospitals' Celebration of the Queen's Jubilee** - 247

**Notes and News.** — Miscellaneous. — Donations. — Queen's Jubilee Proposals.—The Hospitals Association. —Marvellous Calculations.—Four Christmas Days in Hospital.— Vacancies.— The Camberwell Infirmary.— New Hospital for St. Albans.—The Lord Mayor and the Marylebone Infirmary. — Proposed Hospital for Southend.—The "Sister Olive" Question.—Christmas Day at the Oldham Infirmary.—Westminster Hospital. —Hospital Smuggling, etc., etc. - 248

**Christmas Festival at the Hospitals.** By OUR SPECIAL COMMISSIONER - 251

**Christmas Day in a London Hospital.** By a PAYING PATIENT - 252

**Reminiscences of the Insane.** By a MENTAL PHYSICIAN - 253

**Management of the Eye, Ear, and Throat** - 255

**The Editor's Letter-Box.**—

Flowers for Hospitals and Schools - 255

The Story of "Sister Olive" - 256

Glasgow University and Medical School - 256

**Christmas at "The Foundling"** - 256

**Difficult Cases** - 256

**The Children's Corner.**—Puzzles, Answers, etc. - v

**The Hospital Charity Box.**—Prizes and Results - v

